# Non-meningeal, non-pulmonary cryptococcosis with limited posterior uveitis in a kidney organ transplant recipient with antibody-mediated rejection: a case report

**DOI:** 10.1186/s12886-023-03130-w

**Published:** 2023-10-10

**Authors:** Yi-An Lu, Chun-Hsien Lin, Chia-Jen Chang, Kuo-Hsiung Shu, Mu-Chi Chung, Chien-Chih Chou

**Affiliations:** 1 Department of Ophthalmology, Taichung Veterans General Hospital, 1650 Taiwan Boulevard Sect. 4, Taichung, 407219 Taiwan; 2 Division of Nephrology, Department of Internal Medicine, Lin Shin Hospital, No.36, Sec. 3, Huizhong Rd., Nantun District, Taichung City, 40867 Taiwan; 3 Division of Nephrology, Department of Medicine, Taichung Veterans General Hospital, 1650 Taiwan Boulevard Sect. 4, Taichung, 407219 Taiwan; 4 PhD Program in Translational Medicine, National Chung Hsing University, Taichung, Taiwan; 5 Rong Hsing Research Center for Translational Medicine, National Chung Hsing University, Taichung, Taiwan; 6 Department of Medical Laboratory Science and Biotechnology, Asia University, Taichung, Taiwan; 7 Graduate Institute of Clinical Medicine, College of Medicine, National Taiwan University, Taipei, Taiwan; 8 School of Medicine, National Yang Ming Chiao Tung University, Taipei, Taiwan; 9 College of Medicine, National Chung Hsing University, Taichung, Taiwan

**Keywords:** Cryptococcal infection, Solid organ transplant, Kidney transplant, Double filtration plasmapheresis, Immunosuppressant, Uveitis, Choroiditis, Fluconazole, Amphotericin B, Case report

## Abstract

**Background:**

Cryptococcosis is one of the most frequent fungal eye infections in patients with immunosuppression. Currently, treatment approaches for non-meningeal, non-pulmonary cryptococcosis are based on those used for cryptococcal meningitis or pneumonia.

**Case presentation:**

We present a rare case of non-meningeal, non-pulmonary cryptococcosis with clinical manifestations limited to one eye of a cadaveric kidney transplant recipient with chronic-active antibody-mediated rejection. Typical manifestations, diagnosis, and treatments, including antifungal therapies, adjunctive therapies, and immunosuppression reduction, are discussed. After timely diagnosis and treatment, her visual acuity recovered to baseline without recurrence or sequelae of cryptococcosis.

**Conclusions:**

Clinicians should be aware of rare presentations of fungal infections, especially when a kidney transplant recipient with rejection has been treated with intensive immunosuppressants. Early diagnosis with individualized therapies may have a favorable prognosis.

## Background

Immunosuppressants suppress cytotoxic immune reactions to preserve graft function and inhibit local immune reactions to viral, bacterial, or fungal infections. Transplant recipients are vulnerable to various opportunistic infections [1, 2]. Major ocular infection is rare compared with urinary tract infection, pneumonia, or skin and wound site infection in kidney transplant recipients, and is a diagnostic and therapeutic challenge with the possibility of irreversibly damaged vision [1–4].

Cryptococcosis ranks the third most commonly occurring invasive fungal infection in solid organ transplant (SOT) recipients, after Aspergillosis and Candidiasis, and is mostly late-occurring, typically beyond 6 months post-transplantation [5–8]. In a series of transplant patients with cryptococcosis, renal and heart recipients were relatively common [9, 10]. The mean onset time of cryptococcosis infection is earlier in lung (range 8 to less than 12 months) and liver (range less than 12 to 30 months) compared with kidney transplant recipients (range 21 to 48 months) [10–13]. Cryptococcosis frequently presents with central nervous system (CNS) or pulmonary infection, with involvement of other anatomical sites through dissemination, including skin, musculoskeletal system, soft tissue, liver, peritoneum, urogenital tract, adrenals, and eyes. Even if the clinical manifestation is limited to a single anatomical site, non-meningeal, non-pulmonary cryptococcosis generally reflects the consequence of dissemination with similar treatment to that applied for disseminated or CNS diseases [14–16].

We report a rare case of non-meningeal, non-pulmonary cryptococcosis with limited clinical manifestations in one eye of a cadaveric kidney transplant recipient with chronic-active antibody-mediated rejection under intensive immunosuppressants treatment. We also review the literature to evaluate ophthalmic manifestations and treatment modalities.

## Case presentation

**Fig. 1:**
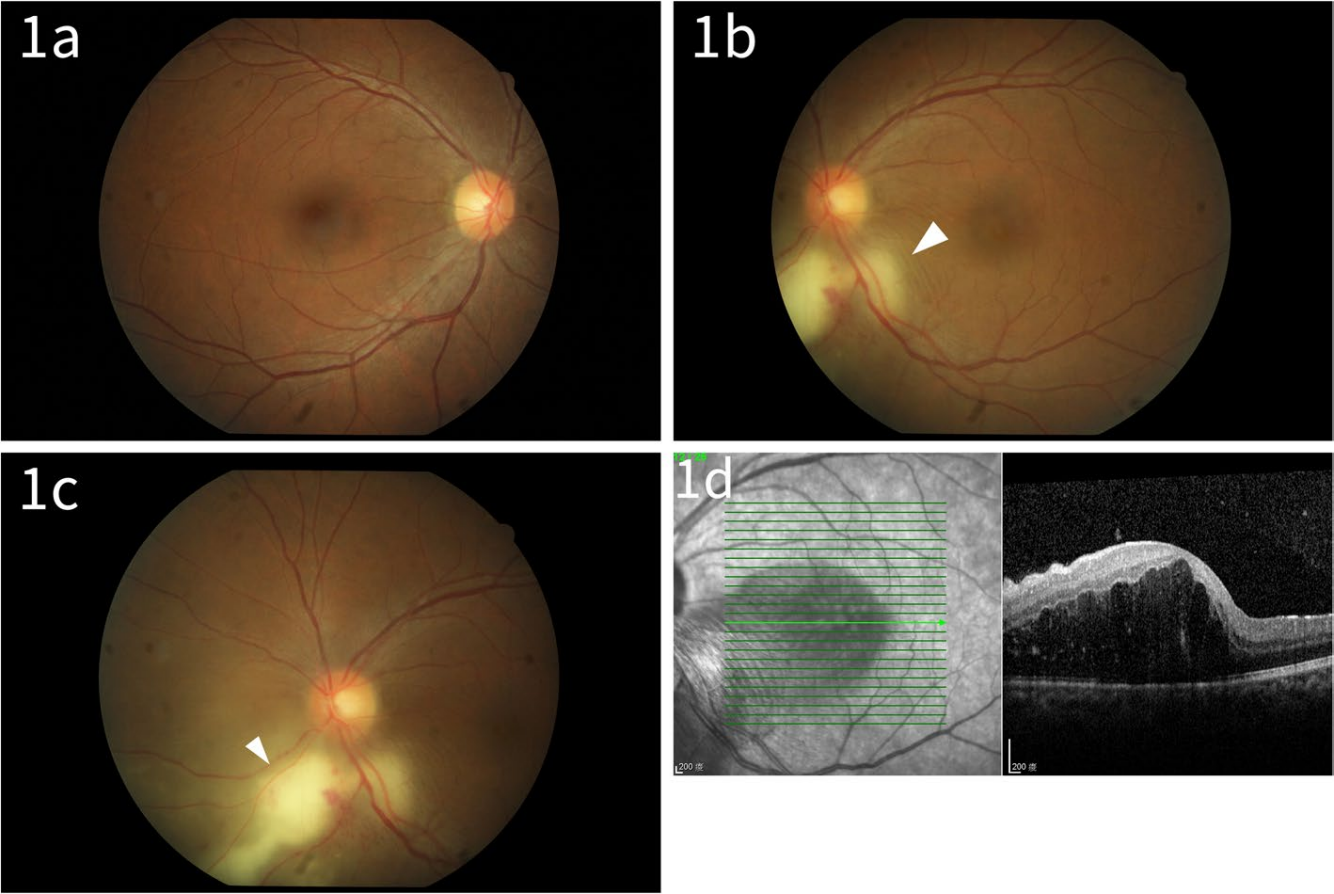
Fundus photographs and OCT images of bilateral eyes on initial presentation: Fundus photographs showed no sign of disease or pathology of right eye (**1a**) while demonstrated peripapillary nasal lower localized choroiditis with exudative retinal detachment (solid triangle) and small multifocal lesions of the left eye (1**b**, 1**c**). OCT image revealed macular edema with subretinal fluid (1**d**)

A 46-year-old Asian woman, with a medical record of psoriatic arthritis (PA), received a cadaveric kidney transplant in May 2009. She developed combined C4d negative chronic-active antibody-mediated rejection (caAMR) in 2015, which was treated with a protocol involving repeated 5 cycles of double filtration plasmapheresis (DFPP) in 2015, 2017, and 2019, along with immunosuppressants (Rituximab 500mg in 2015, three doses of anti-thymocyte globulin 75mg in 2017 and 2019, three doses of Methylprednisolone (MTP) pulse therapy 500mg in 2019). Maintenance immunosuppressants are shown in Table 1. In May 2020, she presented with blurred vision and photophobia in the left eye for 2 weeks upon admission for her fifth DFPP for caAMR, associated with proteinuria, general malaise, and impaired renal function. Her visual acuity was 6/4 in the right eye, 6/5 in the left eye, without specific eye disease in Nov 2019. She had a pet kitten and denied contact or cluster history of pigeon feces, except for her husband's work environment. Apart from the above, a review of systems, especially the CNS and the respiratory systems, was unremarkable. Initial eye examination showed visual acuity of 6/5 in the right eye, 6/60 in the left eye, with intraocular pressures of 19 mmHg in the right eye, 16 mmHg in the left eye. The left eye slit-lamp examination revealed trace anterior chamber cells while fundus examination demonstrated peripapillary nasal lower localized choroiditis with exudative retinal detachment (RD) and small multifocal lesions (Fig. 1b, c). Optical coherence tomography (OCT) showed macular edema in the left eye (Fig. 1d). Examinations of the right eye were unremarkable (Fig. 1a). The preliminary diagnosis was posterior uveitis in the left eye. We suspended Methotrexate and the remaining 2 cycles of DFPP (first 3 cycles were already done), continuously tapered Tacrolimus and Mycophenolate sodium, while keeping prednisolone and salazine at the previous dose. Empirical antimicrobials with intravenous (IV) fluconazole (200 mg/day) and IV ganciclovir (100 mg/day) were prescribed.

**Table 1:** Immunosuppressive agents and FK506 trough level in our case

	Before cryptococcosis	After cryptococcosis
Immunosuppressive agents	Tacrolimus (11mg/day), Mycophenolate sodium (1440mg/day), prednisolone (5mg/day), Methotrexate (5mg/week), salazine (2g/day)	Tacrolimus (7mg/day), Mycophenolate sodium (360mg/day), prednisolone (5mg/day), salazine (2g/day)
FK506 trough level	around 5-6ng/ml	around 3ng/ml

Serology test of toxoplasma-immunoglobulin (Ig) G/IgM, quantiferon-tuberculosis assay, rapid plasma reagin, blood cultures for fungus and bacteria, Epstein-Barr viral capsid antigen-IgM, cytomegalovirus (CMV)-IgG/IgM/quantitative polymerase chain reaction (PCR), herpes simplex virus (HSV)-IgG/IgM, anti-human immunodeficiency virus (HIV) antibody (Ab), and aspergillus galactomannan antigen (Ag) turned out to be negative. Chest X-ray showed no alveolar infiltrates, lymphadenopathy, mass lesions, or pleural effusions, and brain magnetic resonance angiography disclosed no obviously abnormal signal intensity or swelling of bilateral optic nerves. Nevertheless, the blood latex test for cryptococcal antigen (CrAg) was 1:4 initially and elevated to 1:16 two weeks later. Cryptococcal skin lesions were not seen. We shifted fluconazole (applied for the first 4 days) to IV liposomal Amphotericin B (L-AmB) (4 mg/kg/day dissolved in 5% Dextrose). Progressively impaired liver and renal function without specific discomfort developed following three weeks of treatments, with an increase of liver enzymes (AST and ALT) to almost two times the upper limit of normal (ULN) and an elevation of creatinine of about 50% (from 1.96mg/dL to 2.8mg/dL). Moreover, rescue intravenous immunoglobulin (IVIG) (0.4g/kg) was infused once due to hypogammaglobulinemia combined with vital organ involvement.

Lumbar puncture was done to rule out CNS involvement. Cerebrospinal fluid examination revealed a clear fluid without elevated protein level or lymphocytic predominance. India ink stain, latex test for CrAg, gram stain, acid fast stain, cultures for bacteria, virus, fungus and mycobacteria, venereal disease research laboratory tests, antinuclear antibody, adenosine deaminase, anti-double stranded deoxyribonucleic acid (DNA) Ab, IgG index, oligoclonal bands, CMV DNA PCR were all negative. Aqueous humor of the left eye was also collected for PCR of HSV-1, HSV-II, varicella-zoster virus, and CMV, which were all negative.

**Fig. 2:**
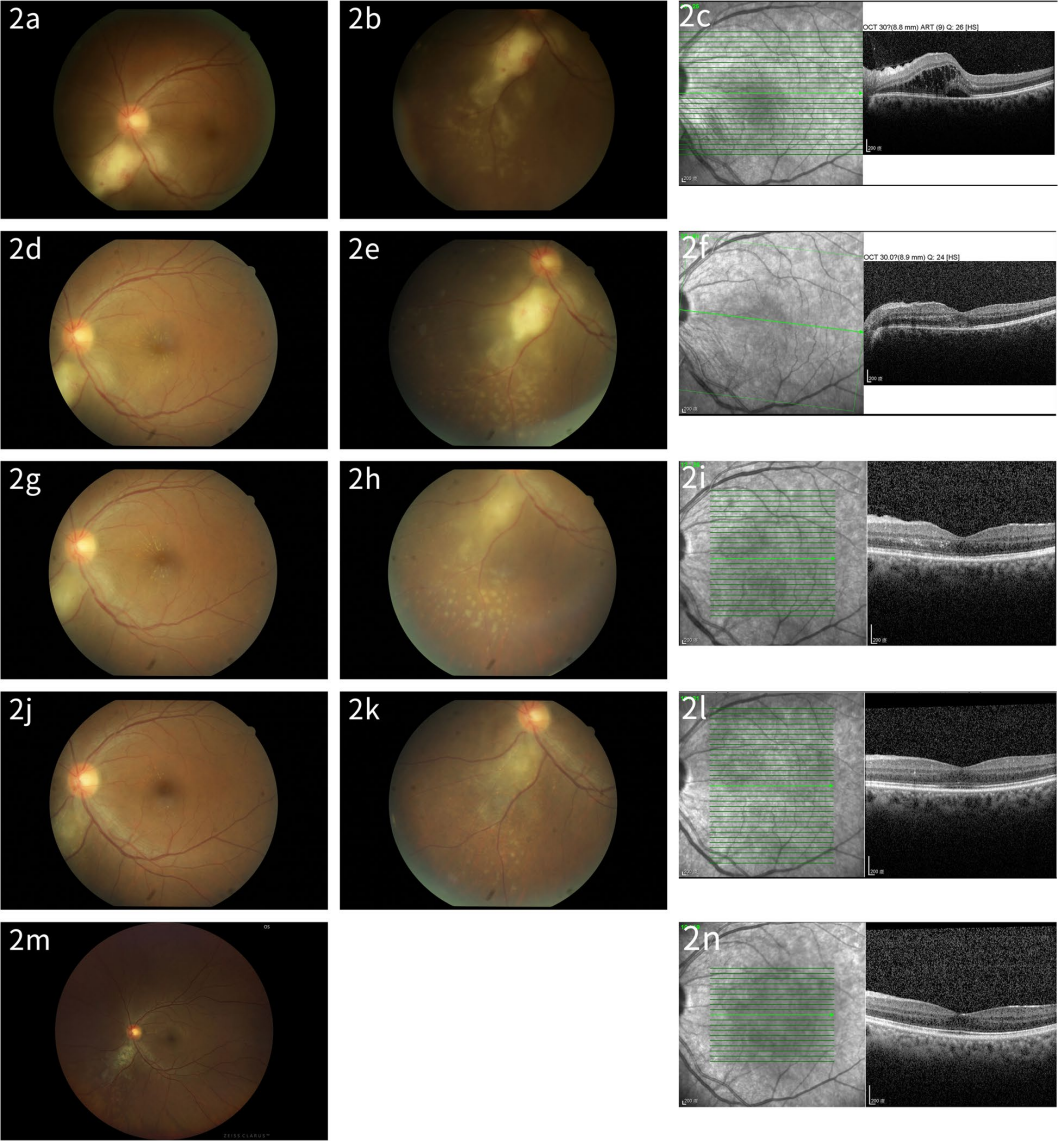
Fundus photographs and OCT images of left eye after treatments: On day 4 after the first visit (post fluconazole and ganciclovir day 4): Fundus photographs (2**a**, 2**b**) showed stable lesions. The OCT image revealed slightly reduced macular edema (2**c**). At 8 days after the first visit (post L-AmB day 4): Fundus photographs (2**d**, 2**e**) showed slightly reduced exudative RD and macular star. OCT image revealed reduced macular edema (2**f**). At 20 days after the first visit (post L-AmB day 16): Fundus photographs (2**g**, 2**h**) showed subsided exudative retinal detachment. The OCT image showed swelling and scarring of the NFL in the lower disc, without macular edema and some HRF in the macula (2**i**). At 1 month after the first visit: Fundus photographs (2**j**, 2**k**) showed post-choroiditis scar and macular star subsided. The OCT image showed that the swelling in the NFL had subsided (2**l**). At 16 months after the first visit: Fundus photographs (2**m**) and OCT image (2**n**) follow-up showed stable conditions

Follow-up examinations (at least 1 time per week) showed deep and silent anterior chamber in bilateral eyes with mild vitritis and choroiditis in the left eye. Fundus demonstrated reduced exudative RD with small multifocal lesion and macular star (Fig. 2a, b, d, e, g, h). OCT revealed markedly decreased subretinal fluid (Fig. 2c, f, i). The left eye visual acuity was improved to 6/15 within three weeks. Due to clinical improvement, L-AmB (totally applied for 3 weeks) was shifted to oral form Fluconazole 200 mg/day with adjusted immunosuppressive agents (Table 1). Her visual acuity recovered to 6/5 in the right eye, 6/6 in the left eye within half a year, and OCT showed no macular edema (Fig. 2j, k, l). Then we finished the treatment (Fluconazole totally applied for about 6 months). Furthermore, her visual acuity improved to 6/5 in the left eye in about 16 months of follow-up (Fig. 2m, n). However, under a relatively low dose of immunosuppressants, her renal function gradually deteriorated. She restarted dialysis in 2023.

## Discussion and conclusions

Ophthalmic symptoms in cryptococcosis range from blurred vision, photophobia, diplopia, nystagmus, ptosis, and ophthalmoplegia to blindness [17–19]. The most common manifestation of ocular cryptococcosis is multifocal chorioretinitis, associated with variable degrees of vitritis, vascular sheathing, exudative RD, papilledema, and granulomatous anterior chamber inflammation [19, 20]. It has been hypothesized that the infection begins as a focus in the choroid, with subsequent extension and secondary involvement of overlying tissues. Ocular infections may occur months after the onset of meningitis or, rarely, before the onset of clinically apparent CNS disease. Outcome of intraocular cryptococcosis was dismal in a previous review, which showed a similar mortality rate to that of cryptococcal meningitis, i.e., about 22% [21].

There are four key components in the management of cryptococcosis in SOT recipients: lumbar puncture for identification of CNS involvement, antifungal therapy, adjunctive therapies, and immunosuppression reduction [11]. The choice of antifungal therapy is typically dependent on sites of infection (meningeal, pulmonary, disseminated or others), severity of illness, immune status, and underlying diseases. As mentioned above, there are no substantial studies evaluating treatment of cryptococcal infection in sites other than lung and CNS in SOT recipients; even for these two sites, treatment recommendations are mainly extrapolated from clinical trials of HIV patients and from data collected retrospectively from SOT recipients [13, 22].

To date, antifungal therapies of cryptococcosis in SOT mainly abide by the revised guidelines of the Infectious Diseases Society of America (IDSA) and the American Society of Transplantation Infectious Diseases Community of Practice (AST IDCOP) (Table 2). Adjunctive therapies, including dexamethasone or Sertraline, are controversial [11]. In general, infection at a single site in the absence of CNS disease or pulmonary disease may be managed with fluconazole (400 mg/d) for 6 to 12 months. Ocular cryptococcosis rarely happens alone, though. When it occurs, therapies require individualization and range from systemic combinations of polyene with high–eye penetration drugs (discussed later) to adjunctive intravitreal AmBd, depending on the extent of eye structure involvement and severities. Blindness and deafness have been reported both in HIV-positive patients and SOT recipients, and some further suggest life-long fluconazole. Even so, fatal outcomes are mainly due to direct involvement of the optic nerve (either by infarction or by cryptococcal infection itself), intracranial hypertension, or late diagnosis and therapies [23].

**Table 2:** Treatment recommendations for CNS or disseminated disease or severe pulmonary disease in transplant recipients^a^

	IDSA, 2010	AST IDCOP, 2019	Our case
Induction therapy	L-AmB (3–4 mg/kg/d) or ABLC (5 mg/kg/d) plus 5-FC (100 mg/kg/d), 2 weeks	L-AmB (3–4 mg/kg/d)^b^ or ABLC (5 mg/kg/d) plus 5-FC (100 mg/kg/d), minimum of 2 weeks	L-AmB 4 mg/kg/d, 3 wks
Alternatives for induction therapy	L-AmB (6 mg/kg/d) or ABLC (5 mg/kg/d) or AmBd (0.7 mg/kg per day), 4–6 weeks	L-AmB (3–4 mg/kg/d) or ABLC (5 mg/kg/d), minimum of 4–6 weeks	
Consolidation therapy	Fluconazole 400–800 mg/d, 8 weeks	Fluconazole 400–800 mg/d, 8 weeks	Fluconazole 200 mg/d, about 6 months
Maintenance therapy	Fluconazole 200–400 mg/d, 6–12 months	Fluconazole 200–400 mg/d, minimum of 6–12 months	

ABLC, amphotericin B lipid complex; L-AmB, Liposomal amphotericin B; AmBd, amphotericin B deoxycholate; 5-FC, 5-fluorocytosine

^a^ Dosages of medicine mentioned above are in the absence of renal insufficiency. All require dose adjustment for renal insufficiency

^b^ Lipid formulation of amphotericin B plus 5-flucytosine is preferred as induction therapy

In our case, whose clinical manifestations exclusively presented with posterior uveitis in the unilateral eye, we applied alternative induction of antifungal therapy without 5-flucytosine (5-FC) to avoid the possibility of bone marrow suppression and nephrotoxicity. CrAg titers generally correlate with initial organism burden and prognosis. However, following titers and the slope of decline do not precisely correlate with clinical response and could not predict recurrence [24, 25]. Some studies showed the lack of 5-FC is an independent risk factor for mycological treatment failure in SOT patients [26–28]. Nevertheless, these circumstances all involve the CNS. With obvious improvement shown both in image and clinical manifestations, we shortened the duration of induction and shifted to a maintenance dose directly.

For tissue penetration of antifungal agents in the eye, fluconazole and 5-FC are detectable in both aqueous and vitreous humors, with and without endophthalmitis, as concentrations are approximately 40% to 100% of those observed in serum. In contrast, ABLC and L-AmB are not detected in non-inflamed eyes, while the penetration rate can be slightly enhanced by inflammation [29]. On the other hand, the choroid and retina are highly vascular compared with the vitreous, and the vascular compartments are separated from intraocular structures by the blood-ocular barrier (BOB). Thus, infection limited to the chorioretinal layers, which are not protected by BOB, is often solely treated with systemic antifungal agents if there are no sight-threatening lesions in the macula [30]. This may explain the outstanding treatment effect of our case with systemic L-AmB and fluconazole. In our patient, eye structure invasion was limited to the choroid and retina. Brain image excluded optic nerve involvement, which made direct extension less likely. After about two weeks of systemic L-AmB, visual acuity was significantly improved.

Immunosuppressant adjustment is of vital importance. Our patient was exposed to a variety of immunosuppressants because of PA and kidney transplantation with caAMR. There is no guideline for immunosuppressant adjustment, though. Antithymocyte globulin and corticosteroids have been associated with an increased risk of cryptococcosis in SOT recipients and all non-HIV infected hosts, respectively [11]. A dose-dependent effect was found in the former [31]. In contrast, calcineurin-inhibitors might not influence the incidence of cryptococcosis and may have anticryptococcal activity because they target the fungal homologs of calcineurin [11]. Treatment of DFPP in combination with other immunosuppressants (rituximab, IVIG, antithymocyte globulin, bortezomib or MTP) in caAMR showed better graft outcome, but a significantly higher rate of adverse events, such as infection and leukopenia [32]. Furthermore, a retrospective cohort study noted a tendency for a higher infection rate with a larger amount of plasma volume by DFPP in late-onset AMR (1–1.3 vs. ≥ 1.3 of total plasma volume) [33]. CMV infection, bacterial pneumonia, and urinary tract infection are major complications in these two studies, with only one case of cryptococcosis in the lung found in the former study. Treatment with rituximab was associated with a high infection rate in SOT recipients, especially in ABO-incompatible renal transplantation recipients. More than 80% of severe infection after rituximab therapy was bacterial infection, followed by viral and fungal infection, with a rare case of cryptococcosis (unknown infection site) [34]. For methotrexate, existing reports have shown controversial results. A case report revealed two cases of cryptococcosis under low-dose methotrexate (10 to 15 mg per week, oral form) for PA and rheumatoid arthritis (RA), respectively [35]. In contrast, a retrospective case–control study found current use of Methotrexate or Sulfasalazine did not significantly increase risk of cryptococcosis among patients with RA [36]. In our case, we kept a lower dose of immunosuppressants after cryptococcosis infection, and there was no ocular recurrence or sequelae of cryptococcosis. However, her renal function gradually deteriorated in the following two years. After an episode of COVID-19 infection in January 2023, her creatinine worsened to 7.1 mg/dL with poor appetite, fatigue, severe hypocalcemia (7.4mg/dL), and hyperphosphatemia (7.6mg/dL). She then started hemodialysis via a previous AV shunt.

In conclusion, a rare case of non-meningeal, non-pulmonary cryptococcosis in a SOT recipient presented with only ocular manifestations. When encountering infectious uveitis in SOT recipients, a rapid diagnosis of cryptococcosis is necessary. Prompt antifungal agents could prevent further vision loss. Complete clinical examinations, especially lumbar puncture and individualized therapies, are of great importance. Treatment goals are eradication of infection and reduced immunosuppressant with preservation of allograft function; however, a dilemma clearly exists in this situation, particularly when organ rejection has already occurred.

## Data Availability

The datasets used and analyzed during the current study are available from the corresponding author on reasonable request, subject to certain conditions and restrictions. The specific location and procedure for accessing the datasets will be provided upon request. The datasets will be available for a specified period of time.

## Abbreviations

Ab: Antibody
Ag: Antigen
BOB: Blood-ocular barrier
caAMR: Chronic-active antibody-mediated rejection
CMV: Cytomegalovirus
CNS: Central nervous system
DFPP: Double filtration plasmapheresis
DNA: Deoxyribonucleic acid
HIV: Human immunodeficiency virus
HRF: Hyperreflective foci
HSV: Herpes simplex virus
Ig: Immunoglobulin
IV: Intravenous
IVIG: Intravenous immunoglobulin
MTP: Methylprednisolone pulse therapy
NFL: Nerve fiber layer
OCT: Optical coherence tomography
PA: Psoriatic arthritis
PCR: Polymerase chain reaction
RA: Rheumatoid arthritis
RD: Retinal detachment
SOT: Solid organ transplant
